# Update on Treatment Guideline in Fibromyalgia Syndrome with Focus on Pharmacology

**DOI:** 10.3390/biomedicines5020020

**Published:** 2017-05-08

**Authors:** Sanam Kia, Ernet Choy

**Affiliations:** 1 Abertawe Bro Morgannwg University Health Board NHS Trust, Neath Port Talbot Hospital, Port Talbot, Wales SA12 7BX, UK; 2 Institute of Infection and Immunity, Cardiff University School of Medicine, Cardiff University, Tenovus Building, Heath Park, Cardiff CF14 4XN, UK; Ernest.choy@wales.nhs.uk

**Keywords:** fibromyalgia syndrome (FMS), guideline, pharmacology, therapy

## Abstract

Fibromyalgia syndrome (FMS) is a chronic condition with unknown aetiology. The pathophysiology of the disease is incompletely understood; despite advances in our knowledge with regards to abnormal central and peripheral pain processing, and hypothalamo–pituitary–adrenal dysfunction, there is no clear specific pathophysiological therapeutic target. The management of this complex condition has thus perplexed the medical community for many years, and several national and international guidelines have aimed to address this complexity. The most recent guidelines from European League Against Rheumatism (EULAR) (2016), Canadian Pain Society (2012), and The Association of the Scientific Medical Societies in Germany (AWMF) (2012) highlight the change in attitudes regarding the overall approach to FMS, but offer varying advice with regards to the use of pharmacological agents. Amitriptyline, Pregabalin and Duloxetine are used most commonly in FMS and though modestly effective, are useful adjunctive treatment to non-pharmaceutical measures.

## 1. Introduction

Fibromyalgia syndrome (FMS) is a chronic condition characterised by generalized body pain, fatigue, sleep disturbance, impaired cognition, and anxiety with unknown aetiology. Potential causes include genetic, neurologic, psychologic, sleep and immunologic factors [1]. Fibromyalgia has an estimated prevalence of 0.5% to 5.8% in North America and Europe [2]. The pathophysiology of the disease is not clearly understood, although abnormality in pain processing at various levels (peripheral and central), sleep impairment, dysregulation of the hypothalamo–pituitary–adrenal axis, and abnormalities of the autonomic nervous system have been identified as contributory factors. Despite our increased understanding of the condition, there are no objective diagnostic tests. Diagnosis is often made by exclusion of other conditions such as neurological syndromes and depression. This lack of a single unifying pathophysiology is mirrored by a complex and non-specific approach to management.

The first clinical criteria for the diagnosis of FMS was set in 1990 by the American College of Rheumatologist (ACR) [3]. It was based on widespread body pain (defined as the pain affecting both sides of the body above and below the waist) for at least three months plus tenderness in at least 11 out of 18 tender points. In 2010, these criteria were updated to change the focus towards a subjective widespread body pain index (WPI) and symptom severity scale (SS), taking into account cognitive symptoms, sleep, fatigue and additional somatic symptoms [4].

Despite our greater understanding of the disease, there is no definitive treatment for FMS and various guidelines for treatment exist, which are at time contradictory in their advice. The approach to managing FMS has evolved over recent years, as reflected by recently updated guidelines published by European League Against Rheumatism (EULAR) [5]. Increasingly, there is a focus on non-pharmacological therapies for FMS, such as cognitive behavioural therapy, exercise therapy, hydrotherapy, and acupuncture. Although these advances may have aided the management of FMS, providing clinicians and patients with alternative therapy avenues to pursue, pharmacology remains the mainstay of therapy. Therapeutic classes and targets of pharmacologic therapy in FMS are varied, including classical analgesic therapies such as opiates, and ranging to antidepressants, anticonvulsants, and others. This wide range of therapies often leads to confusion in the clinic, and the evidence supporting one therapy over another is limited. This is reflected in the guidance, which offers evidence for the potential utility of each class of pharmacological intervention, but does not necessarily support one form over another and certainly does not provide a treatment hierarchy. Compounding these difficulties, there is also a significant degree of variability between the guidance.

In this paper, we will review the most recent national and international guidelines for the management of fibromyalgia, with a particular focus on the pharmacological agents. We will set out the evidence for the use of each class of pharmacological agent derived from each guideline, and form a synthesis of the evidence with the aim of assisting clinicians in gaining an overview of each intervention based on the agent, rather than divided by guideline. The process and quality of the guideline drafting process will also be assessed.

## 2. The Guidelines

The EULAR 2016 guidelines [5] were developed by 18 members from 12 European countries including clinicians, no-clinical scientists, patient representatives, and allied health professions. The guideline is based upon synthesis of systematic reviews (with or without meta-analysis) with pain as the primary outcome, although fatigue, sleep, and daily function were also used as secondary endpoints. The authors used the Grading Recommendations Assessment, Development, and Evaluation system (GRADE) for making recommendations, with the strength of recommendations based on desirable and undesirable effects. This is a four-point scale: strong for/weak for/weak against/strong against (Table 1). All participants then voted on the level of agreement. In their 10 recommendations, exercise therapy has been given the highest level of recommendation which is a stark difference from the previous guideline [6] where pharmacological treatment was considered to be the focus of therapy. Initial management should involve patient education and focus on nonpharmacological therapies. In non-responsive patients, pharmacological therapies should be added especially to those with sleep or mood disturbances.

The 2012 Canadian guideline [7,8] was developed by the Canadian Fibromyalgia Guideline Committee (CFGC), comprising of 12 members of relevant healthcare professionals, a patient representative, an external international expert and a research coordinator. The literature search covered a 20-year period (1990–2010) and a broad scope of evidence was considered not limited to randomised controlled trials (RCTs). The recommendations were then submitted to the 35 members who form the National Fibromyalgia Guideline Advisory Panel. Recommendations were graded according to the classification system of the Oxford Centre for Evidence Based Medicine [9]. The primary recommendation of these guidelines indicates a paradigm shift in terms of the diagnosis and management of fibromyalgia, with an emphasis on the delivery of care in the community. Greater patient involvement in terms of educations and self-management was also highlighted. The guideline acknowledges that pharmacological therapy, even at best, is only modestly effective, but that regular physical activity should be considered the cornerstone of treatment, with the highest level of recommendation.

The German guideline [10] was developed by the Association of the Scientific Medical Societies in Germany (AWMF) which is a 12-member committee comprising of representatives from 10 scientific societies and two patient self-help organizations. The process was initiated and coordinated by the German Interdisciplinary Association of Pain Therapy (DIVS). The recommendations are based on a systematic review of the literature (all controlled studies, systematic reviews and meta-analysis) up to 31 December, 2010. In the AWMF guidelines, where no high-quality evidence was reported to be available, uncontrolled trials and case series were reviewed and expert opinion was sought. Level of evidence was based on the Oxford Centre for Evidence Based Medicine system [11], and the grading of the recommendations based on the German Programme for Disease Management guidelines [12] (Table 1). The recommendations are made based on the efficacy of the intervention, associated risks, patient preference and practicability/applicability (i.e., was the drug approved for therapy of FMS and/or its common comorbidities in German). The AWMF guideline highlights the importance of partnership with patients and also developing realistic aims of therapy based on informed-decision making, local availability, cost and patient presences. The summary recommendation is strongly in favor of physical activity treatment options, such as exercise and psychological therapies. Continuous pharmacological treatment is only recommended where the benefits are sustained. 

The comparison of the categorisation of the evidence and recommendations of the three guidelines is listed in Table 1.

## 3. Pharmaceutical Therapies

Each of the three guidelines was interrogated for its source data, and the levels of evidence for each pharmaceutical agent/class evaluated based on the included articles. Unless stated, the guidelines used systematic review articles and meta-analysis as the basis of their recommendations, although the robustness of these reviews and the quality of the included studies was variable. The summaries of the article are included in Appendix A. The choice of pharmaceutical therapies in treating patient with FMS should be guided by the patient’s clinical features, side effect profile, and response. FMS patients started on pharmaceutical therapies should be reviewed frequently and the dose titrated up based upon the patient’s response. Where therapy has not demonstrated a positive effect, or where side effects are prominent, treatment should be discontinued.

## 4. Amitriptyline

Amitriptyline (AMT) is a tricyclic antidepressant known to inhibit both serotonin and noradrenalin reuptake, and has long been used for the management of neuropathic pain and FMS. The AWMF guidelines assessed 12 studies in their meta-analysis, whereas the Canadian guidelines examined only two systematic reviews, whilst EULAR examined five review articles (Table A1, Appendix A).

AMT has received a strong recommendation from AWMF (10–50 mg/daily), while the EUALR guidelines suggest only low dose may be beneficial, although with a high degree of consensus. The Canadian guidelines take a much more a general approach to AMT, and recommend that all categories of antidepressants, including SSRI and NSRI may be used for the treatment of FMS depending on the individual efficacy of the drug, physician’s knowledge, patient’s characteristics, and cost. Nishishinya et al. also concluded that AMT 25 mg/day improved pain, sleep and fatigue at 6–8 weeks with no evidence for 50 mg/day [13].

## 5. Anticonvulsants

Pregabalin (PGB) and Gabapentin (GBP) are both structurally similar to the neurotransmitter γ-aminobutyric acid (GABA), although neither drug has activity in GABA’s neuronal system. Their analgesic effect is linked to their ability to bind to voltage-gated calcium channels in the central nervous system (CNS) [14]. An eight-week, double-blind, randomised clinical trial of 529 patients with FMS demonstrated the efficacy of PGB at different doses (300 mg, 450 mg), demonstrating a more than 50% improvement in pain with PGB 450 mg/day. Other domains including sleep quality, fatigue, and health related quality of life also showed improvement [15]. In a Cochrane review, a daily dose of 600 mg produced no better outcome than 450 mg/day for any outcome measures [16]. The recommendations across the guidelines with regards to the use of these drugs are somewhat variable; the CPS recommends using anticonvulsants with data derived from level 1 evidence (Table A2, Appendix A), while AWMF recommends using PGB (150 to 450 mg/day) if treatment with AMT is not possible, and refrains from making any recommendations with regards to GBP. EULAR guideline analyses nine review articles on PGB (*n* = 1481 to 3334) and one clinical trial on gabapentin (*n* = 150). EULAR recommends use of PGB (weak for), and gabapentin for research purposes only.

Use of PGB and GBP may be limited by their side effect profile such as dizziness, somnolence, weight gain, peripheral oedema, and negative neurocognitive effects [17].

## 6. Serotonin–Noradrenalin Reuptake Inhibitors (SNRI)

Serotonin (5-HT) and noradrenalin have been implicated in the mediation of the descending pain inhibitory pathways [18], which have in turn been linked to the pathophysiology of FMS. Patients with FMS have been found to have decreased concentration of 5-HT and its precursor (tryptophan) in serum and cerebrospinal fluid [19]. Serotonin is implicated in psychiatric disorders such as depression and anxiety [20], and is theorized to have a role in pain threshold and stage 4 sleep [19].

Duloxetine (DLX) has a five-fold stronger effect on serotonin than on noradrenalin [21]. AWMF analyses five RCTs with 1157 participants, whilst EULAR uses eight systematic reviews with 443 to 2249 participants (Table A5, Appendix A). AWMF recommends DLX (60 mg/day) for patients with comorbid depressive disorder, with or without general anxiety disorders. This recommendation is also endorsed in the CPS and EULAR guidelines. DLX dose and length of therapy is guided by patient response and side effect profile. However, DLX 20–30 mg/day has not shown to be effective, and no difference was found between 60 mg/day compared to 120 mg/day [22].

Milnacipran (MLN) has three-fold stronger effect on noradrenalin than serotonin. It is recommended by EULAR (seven systematic reviews) and has been shown to be effective [21,23,24,25,26], though DLX was found to be superior to MLN in reducing pain and sleep problems [27]. AWMF guidelines do not recommend the use of MLN. This is based on low quality evidence, with low acceptance amongst patients and high risks of side effects. There is not enough available evidence with regards to the use of other agents such as venlafaxine in the management of FM.

## 7. Selective Serotonin Reuptake Inhibitors

A recent Cochrane review concluded that there was no unbiased evidence with regards to superiority of SSRIs to placebo in treating the key symptoms of fibromyalgia (pain, fatigue and sleep problems), however they might be considered for treating depression in this group of patients [28]. National and international guidelines are mixed with regards to their recommendations on SSRIs. EULAR guidelines are derived from seven systematic reviews, whilst AWMF uses eight RCTs in their meta-analysis (Table A4, Appendix A). EULAR does not recommend their use, whereas the Canadian and AWMF guidelines do recommend their use. Fluoxetine 20–40 mg/day or paroxetine 20–40 mg/day can be considered for a limited period of time in comorbid depressive/anxiety disorders [29,30]. Citalopram was ineffective in management of FMS in a small RCT of 40 patients [31].

## 8. Opioids

Use of strong opioids has been discouraged in the treatment of FMS. There is a deficit in opioid mediated descending anti-nociceptive activity in patients with FMS, with increased level of endogenous opioids in the CSF [32] and decreased central μ-opioid receptor availability [33], which may explain the lack of effectiveness of exogenous opioids in this group of patients.

Tramadol is a weak opioid with combined μ-receptor agonist and 5-HT and norepinephrine reuptake inhibition activity [34]. It is this latter action that is possibly the key in its efficacy in FMS compared to other opioids. The efficacy of tramadol in FMS has been studied in number of trials [35,36,37,38], although the long-term efficacy and the optimal dose of tramadol have not been addressed by the clinical trials. EULAR guidelines use two meta-analysis, Canadian guidelines 2RCTs whilst AWMF uses only one RCT (Table A6, Appendix A). Tramadol is recommended by EULAR and the Canadian guidelines, whereas AWMF refrain from making any recommendations on the basis of lack of data.

## 9. Cyclobenzaprine

Cyclobenzaprine is a centrally acting muscle relaxant which is structurally related to TCA, and which was first developed as an antipsychotic therapy [39]. The EULAR guideline recommends the use of cyclobenzaprine (weak for, 75% agreement) based on one systematic review involving 312 patients (Table A3, Appendix A) [40]. Overall, patients treated with cyclobenzaprine were three times more likely to report overall improvement but there was no improvement on fatigue. In total, 85% of patients experienced side effects and only 71% completed the studies. The AWMF guidelines do not recommend the use of this medication on the basis of lack of license for its use, and risk of side effects (confusion, skin lesions, liver toxicity).

## 10. Cannabinoids

Cannabinoid molecules have been shown to have analgesic properties as well as sleep promoting affects [41], hence there has been increasing interest in their use in pain management. The endogenous cannabinoids and their receptors have been localized to multiple levels of nervous system (both peripheral and central) [42], and have an acknowledged role in helping to regulate neural process associated with pain perceptions, mood, appetite and memory [43]. The Canadian guidelines recommend the use of pharmacological cannabinoids, particularly in the setting of sleep disturbance, although it has not received any recommendations by AWMF and EULAR. There is also concern with regards the risk of abuse [44], there is a general need for further research into their use.

## 11. Analgesic Treatments (Non-Steroidal Anti-Inflammatory (NSIADs) and Acetaminophen)

Use of NSIADs for the management of FMS symptoms are not recommended by EULAR and AWMF. These recommendations come from a small number of studies (Table A7 Appendix A). A recent Cochrane review on NSAIDs in FMS also came to the same conclusion [45]. However, the Canadian guidelines, though not directly supporting their use in FMS, recommend using this group of drugs with the lowest possible dose for the shortest possible times in concurrent conditions such as osteoarthritis. Acetaminophen’s actions, such as modulating the endogenous cannabinoid system [46], and serotonin receptor agonist [47], may be beneficial in FMS. There is no direct evidence with regards to the use of acetaminophen in FMS though it has been used in combination with tramadol [35].

Table 2 provides the list of medications that are not recommended for use in FMS, and which are not discussed in this paper.

## 12. Discussion

Discordance between the guidelines on recommendations for pharmacological treatments for of FMS is primarily due to lack of high-quality randomized control trials in FMS, hence the guidelines rely on lower-quality evidence and expert consensus. Perhaps most tellingly, the latest guidelines all recommend non-pharmacological therapies, such as exercise, with a greater level of confidence than pharmacological agents, perhaps in part due to low risk of side effects and the generic health benefit of exercise. Despite this, the pharmacological treatment of FMS has an acknowledged role, especially where symptoms of or a concurrent diagnosis of depression is prominent.

One potential source of divergence in the guidelines in in the difference between their respective committees. The EULAR committee is formed of experts from 12 European countries with different health care systems, populations needs, and to some degree availability of therapies. EULAR also focuses mainly on systematic reviews (with or without meta-analysis), which provide the highest level of evidence, although narrowing the breadth of the guidance. AWMF, however, uses clinical trials as well as the systematic reviews, thus potentially capturing a greater number of admittedly lower quality studies. The Canadian guidelines uses original studies, systematic reviews, and evidence-based guidelines, and was perhaps the broadest approach to evidence gathering, although the number of included studies was actually less than that of the AWMF guidelines.

Further to the methodological differences between the guidelines, the divergence of the guidelines is also compounded by the variability with regards to the licensing of drugs in FMS. In the US, the FDA approved medications for FMS include duloxetine, MLN, and PGB, whereas opioids are not approved for treatment of fibromyalgia. Only PGB and duloxetine had Health Canada approval for management of FMS, and the use of other drugs is effectively “off-label” (use the guidance ref on line). In Germany, no drug is specifically licensed for FMS. Each of these factors may influence the prescribing patterns of clinicians managing patients included in the contributory data, and in the future of clinical practice.

Additionally, the guidelines do not offer information based on the outcomes for each of the six OMERACT core domains (pain, sleep disturbance, fatigue, affective symptoms, functional deficit, and cognitive impairment) [48], which would have allowed direct comparison of the evidence between studies and pertaining to individual disease components. This analysis would in turn help clinicians provide individualized care for patients exhibiting differing manifestations of FMS.

The guidelines are however all in agreement that therapy should be tailored to the individual needs of the patient, and that non-pharmacological therapy should be encouraged in the first instance. This is again reiterated in the Canadian guidelines, where a shift to community based care (general practice) is emphasized, also highlighting the fact that the ACR diagnosis guidelines (1990 and 2010) were mainly designed for research purposes.

EULAR recommends using DLX, PGB, or tramadol (in combination with paracetamol) in severe pain, and that AMT, cyclobenzaprine, or PGB should be considered at night for sleep problems. This is in contrast to the Canadian guidelines, which make only general recommendations on groups of drugs, rather than focusing on a specific agent. Despite the guidelines stating level 1 evidence for antidepressant medications (TCA, SSRI, and SNRI) and anticonvulsants, it does not provide enough information to the prescriber with the regards to the choice of medication, optimum dosage, and the duration of therapy.

The AWMF (2012) guidelines recommend AMT as a first-line pharmacological treatment, with duloxetine as a second-line treatment in patients who cannot tolerate AMT and have concurrent depressive disorder. The AWMF guidelines are also more explicit with regards the use and duration of the pharmacological therapy; recommending a trial treatment of at least 6 months, after which, if no effect is observed, the therapy should be stopped. This is based on the maximum duration of RCT study with AMT, Duloxetine and Pregabalin being 6 months [10].

Canadian 2012 guidelines, as well as shifting the diagnosis and management of FMS to the primary care, support the use of combination of non-pharmacological and drug therapy in fibromyalgia. However, their recommendation with regards to the pharmacological therapy is vague and depends heavily on the expertise of the reader.

Despite advances in the understanding of the pathophysiology of FMS, the pharmacological management has remained complex and poorly evidenced. What is clear is that an individualized approach to patient care is imperative, and that pharmacological therapy should be considered as an adjunct to non-pharmacological intervention. Where medication is deemed necessary, it should be targeted specifically towards the specific symptoms of the FMS in the individual patient, and if no improvement is observed after a reasonable trial period, should be discontinued. It is also important to note the risks of side effects of these medications, which may add to the complexity of the picture (Table 3). There is currently no evidence to support the use of multiple therapies for FMS, although this is commonly seen in clinical practice. Concerns regarding licensing should be addressed by regulatory bodies, especially where there are concerns regarding the widespread use of “off-license” therapies.

## Figures and Tables

**Table 1:** Comparison of the categorization of evidence and recommendations of the four guidelines used for therapy/prevention/aetiology and adverse reactions.

Level of Evidence/Recommendation	AWMF 2012	Canadian Guideline 2012	EULAR 2016
Evidence level I	Ia—SR (with homogeneity) of RCTs Ib—individual RCTs (with narrow confidence intervals) Ic—All or non	Systematic review of randomised trials or n-of-1 trials (treatment) Systematic review of inception cohort studies (outcome)	RCTs (double blind)
Evidence level II	IIa—SR (with homogeneity) of cohort studies IIb—single cohort studies (including low quality RCT; e.g., <80% follow up post observation rate) IIc—results search; ecological studies	Randomised trial or (exceptionally) observational study with dramatic effect (treatments) Inception cohort studies (outcome)	Randomised double blind crossover trials
Evidence level III	IIIa—SR (with homogeneity) of case-control studies IIIb—single case-control studies	Non randomised controlled cohort/follow-up study (treatment) Cohort of control arm of randomised trial (outcome)	Randomised single blind trials
Evidence level IV	Case series and poor quality cohort and case-control studies	Systematic review of case control studies or historically controlled studies (treatments) systematic review of case series	Randomised open trials
Evidence level V	Expert opinion without critical analysis	Opinion	Non randomised open trials
Recommendations			
Strength A	Based on evidence level 1(subject to up/downgrade)	Consistent level I studies	Strong for
Strength B	Based on evidence level 2 (subject to up/downgrade)	Consistent level II Or III studies, or extrapolations from level 1 studies	Weak for
Strength C	Based on evidence levels 3,4,5 (subject to up /downgrade)	Level IV studies or extrapolations from level II or III studies	Weak against
Strength D	-	Level V evidence or troubling inconsistent or inconclusive studies of any level	Strong against
Panel consensus	-	Opinion supported by entire Canadian Fibromyalgia Guidelines Committee	-

Abbreviations: SR = systematic review, RCT = Randomised Control trial.

**Table 2:** Medications not recommended for treatment of fibromyalgia. Where no recommendation for/against is offered, a grey box is used. LE: level of evidence.

Drug	AWMF (LE)	EULAR	Canadian Guideline
Acetaminophen	No positive or negative recommendation		May be used in some patients (level 5)
Antiviral Drugs	Strong negative (2b)		
Anxiolytics	Strong negative (2b)		
Dopamine agonists	Strong negative (2)a		
Flupirtine	Negative (4)		
Hormones (Growth hormone, Glucocorticoids, Calcitonin, oestrogen)	Strong negative (3a)	Strong against	
Hypnotics	Strong negative (3a)		
Interferon	Strong negative (3a)		
Ketamine	Strong negative (4a)		
Local anaesthetic	Strong negative (3a)		
Monoamine oxidase inhibitor	Negative (2a)	Weak against	
Sodium Oxybate	Strong negative (3a)	Strong against	
Neuroleptics	Strong negative (3a)		
Strong opioids	Strong negative (4b)	Strong against (5)	Discouraged Level 5, grade D
Serotonin Receptor Antagonist	Strong negative (3a)		

**Table 3:** Contraindications, warning and precautions with regards to the most commonly used drugs in fibromyalgia syndrome (FMS) as per summary of product characteristic (SPC).

Drug	Contraindications	Warning and Precautions	Last Update
Amitriptyline	Prior Hypersensitivity Concomitant use of MAOI Acute recovery phase following myocardial infarction Mania Sever liver disease Congestive heart failure	Suicidality Hyponatraemia QT interval prolongation on ECG Blood dyscrasias	5 December 2016
Duloxetine	Serotonin syndrome and MAOIs. Concomitant use of irreversible MOAi, fluvoxamine, ciprofloxacin or enoxacin Liver disease resulting in hepatic impairment Severe renal impairment	Mania and seizures Mydriasis Hypertension Renal impairment Serotonin syndrome Suicide Diabetic peripheral neuropathic pain Hyponatraemia	8 February 2008
Pregabalin	Known hypersensitivity to pregabalin (PGB) or any of its components	-Hypersensitivity reaction -Dizziness, somnolence, loss of consciousness -Vision-related effects -Increase risk of suicidal thoughts and behaviours -Encephalopathy Reduced lower gastrointestinal tract function	14 November 2016
Tramadol Hydrochloride	Hypersensitivity to tramadol or other opioids Severe hepatic/renal impairment MOA or within 2 weeks of their withdrawal	Withdrawal symptoms Dependence and abuse Convulsive disorders	22 September 2015
Milnacipran	HypersensitivityConcomitant use to MAOI Liver disease resulting in hepatic impairment Uncontrolled hypertensionSevere renal impairment	As per Duloxetine	8 February 2017
SSRI (Fluoxetine)	Concomitant of metoprolol and irreversible non-selective MAO, hypersensitivity to the active substance	Suicidality Rash and allergic reaction Seizures Mania Hepatic/renal function Prolonged QT	22 December 2016

Abbreviation: MAOi = monoamine oxidase inhibitor, SSRI = selective serotonin reuptake inhibitor.

## Appendix A

List of evidence used to formulate the guidelines.

**Table A1:** Amitriptyline (AMT).

Guidelines	Type of Studies	Studies	Comment	Recommendation
EULAR	5 Reviews (5–13 clinical trials, 322 to 919 participants)	Häuser, W. et al. 2011 [49]	Systematic review with meta-analysis including 5 trials (*n* = 612). AMT cannot be regarded as the gold standard of FMS therapy with antidepressants because of the methodological limitations of its trials.	AMT Weak for, at low dose (100% agreement)
		Nishishinya, B. et al. 2008 [13]	Systematic review including 5 RCT s (*n* = 615). Definitive clinical recommendation regarding the efficacy of AMT for FM could not be made. There is some evidence to support short-term efficacy of AMT 25 mg/day. However, there is no evidence to support higher dose or for period more than 8 weeks.	
		Üceyler, N. et al. 2008 [50]	Systematic review including 13 RCTs (*n* = 850). AMT 25–50 mg/day reduced pain, fatigue and depression in patients with FMS with improvement in sleep and quality of life.	
		Moore, R.A. et al. 2012 [51]	Cochrane review including 7 RCTs (*n* = 548). There was some evidence that amitriptyline at 25 or 50 mg daily was better than placebo.	
		Perrot, S. et al. 2014 [52]	Meta-analysis, including 5 studies (*n* = 500). AMT was effective at reducing pain, sleep disturbance and improving fatigue.	
AWMF	18 systematic reviews were analysed overall, (*n* = 1014) with 14 studies on AMT. 12 studies were included in the meta-analysis which are listed in this table.	Capaci, K. et al. 2002 [53]	Randomised trial, paroxetine (20–40 mg/daily) vs. AMT 10–20 mg daily. AMT works better than paroxetine.	AMT 10–50 mg/day should be used for a limited period of time. Level of evidence 2a. Strong consensus.
		Carette, S. et al. 1986 [54]	Double blind placebo control trial, comparing AMT 50 mg/day to placebo. Improvement in sleep and physician global assessment in the MT group.	
		Carette, S. et al. 1994 [55]	Randomised double blind trial comparing AMT, cyclobenzaprine and placebo confirmed short-term efficacy of the two drugs in FMS.	
		Carette, S. et al. 1995 [56]	Double blind, cross-over trial of AMT (25 mg) vs. placebo. Alpha NREM abnormality exists in a small group of patients with FM and is not corrected by AMT.	
		Caruso, I. et al. 1987 [57]	Double blind study of dothiepin versus placebo which showed improvement in the treatment arm.	
		Garcia, J. et al. 2006 [58]	Randomised controlled trial showed that CBT was superior to pharmacolical therapy (cyclobenzaprine) in FMS.	
		Ginsberg, F. et al. 1996 [59]	Randomised placebo-controlled trial on use of sustained released AMT (25 mg) showed potential benefits in FM.	
		Goldenberg, D.L. et al. 1986 [60]	Randomised controlled trial of AMT (25 mg) and Naproxen, which showed AMT was associated with significant improvement in all outcomes.	
		Goldenberg, D. et al. 1996 [29]	Randomised double blind cross-over trial of AMT (25 mg) and Fluoxetine (20 mg) showed both are effective and they work better in combination in FMS.	
		Hannonen, P. et al. 1998 [61]	Randomised double blind placebo controlled study on AMT and moclobemide showed that AMT was effective in FMS (general health, pain and sleep quality).	
		Heymann, R.E. et al. 2001 [62]	Randomised double blind control study on AMT (25 mg), nortriptyline (25 mg) and placebo. All three groups improved. Only the patients’ subjective global assessment differed between the AMT group.	
		Kempenaers, C. et al. 1994 [63]	Randomised double blind trial comparing SER 282 with AMT (50 mg) or placebo. AMT did not have any effect on sleep.	
		Scudds, R.A. et al. 1989 [64]	Randomised double blind cross-over study on AMT (25 mg–50 mg) vs. placebo. AMT was associated with significant improvement.	
CANADA	2 systematic reviews	Hauser, W. et al. 2009 [65]	7 studies, *n* = 446 , Strong evidence for the efficacy of the amitriptyline in reducing pain, fatigue and sleep disturbances It concludes that short-term usage of AMT can be considered for treatment of pain and sleep disturbance in FMS.	TCA may be used in FMS.
		Üceyler, N. et al. 2008 [50]	As Per EULAR	Level 1, Grade A

**Table A2:** Anticonvulsants.

Guideline	No. Reviews	Reviews	Comment	Recommendation
EULAR	PGB 9 reviews (1481 to 3334 participants) Gabapentin 1 clinial trial 150 participants—not relevant	Choy, E. et al. 2011 [66]	Systematic review and mixed treatment comparison. 5 RCTs on Pregabalin confirmed therapeutic efficacy (pain, fibromyalgia impact questionnaire scores) for PGB.	PGB weak for (94% agreement) Gabapentin—research only (100% agreement).
		Tzellos, T.G. et al. 2010 [67]	Systematic review and meta-analysis on gabapentin and PGB. 4 RCTs (*n* = 2040) PGB at the dose of 450 mg is most likely effective in treating FM, the data on gabapentin is limited. Adverse events are not negligible	
		Üceyler, N. et al. 2013 [68]	Cochrane review PGB demonstrates a small benefit in reducing pain and sleeping problems with no substantial effect on fatigue compared to placebo. Study drop-out rates due to adverse events were higher with PGB use compared with placebo.	
		Siler, A.C. et al. 2011 [69]	Systematic review, 5 RCTs on PGB and 1 on gabapentin. PGB and gabapentin are modestly effective in treatment of fibromyalgia though their long-term safely and efficacy remains unknown.	
		Roskell, N.S. et al. 2011 [70]	Meta-analysis, PGB 3 RCTs, Gabapentin 1 RCT. A 30% pain reduction was observed in patients treated with gabapentin and PGB (300 mg and 450 mg). There was also significant risk of discontinuation due to adverse events.	
		Perrot, S. et al. 2014 [52]	Meta-analysis, including 2 studies on PGB (*n* = 1497) showing efficacy in pain relief and improvement of function.	
		Häuser, W. et al. 2009 [17]	Meta-analysis on treatment of FM with gabapentin and PGB. There was strong evidence for reduction of pain, improve sleep and quality of life but not for depressive symptoms.	
		Moore, R.A. et al 2009 [16]	Cochrane review, on PGB for acute and chronic pain. Pregabalin was effective at doses of 300 mg, 450 mg and 600 mg in treating of variety of pain conditions including FMS (5 studies).	
		Häuser, W. et al. 2010 [21]	Comparative efficacy and harms of DLX, MLN, in PGB in FMS. The three drugs are superior to placebo except DLX for fatigue, MLN for sleep disturbance and PGB for depressed mood.	
AWMF	4 RCTS, *n* = 4132	Crofford, L.J. et al. 2005 [15]	Multi-centre double blind RCT on 529 patients. PGB at 450 mg/day was efficacious for the treatment of FMS, reducing symptoms of pain, disturbed sleep, and fatigue compared with placebo. PGB was well tolerated and improved global measures and health-related quality of life	Treatment with PGB (150–450 mg/day) can be considered for a limited time period, if treatment with AMT is not possible. Level of evidence 1a, open recommendation. Due to the limited data available for gabapentin neither a positive nor a negative recommendation is possible. Strong consensus.
		Arnold, L.M. et al. 2008 [71]	Randomised double blind placebo controlled trial of PGB 300 mg, 450 mg and 600 mg/day showed that all three doses were efficacious for up to 14 weeks.	
		Mease, P.J. et al. 2008 [72]	Randomised double blind placebo controlled trial on the use of PGB 300, 450, and 600 mg/day which was efficacious and safe in the treatment of pain in FM.	
		Pfizer (not published at the time the guidelines was written)	14 week randomised, double blind placebo controlled trial of Pregabalin twice daily in patients with FM.	
		Arnold, L.M. 2007 [73]	Randomised double blind, placebo-controlled trial, Gabapentin is effective and safe in FM.	
Canada	3	Holtedhal. 2010 6 reviews [74]	(Article in Norwesian) review of 6 randomised double blind controlled trial. Recommendations for PGB is based on rather weak evidence. Mean pain reduction between 9% to 15%.	Treatment with anticonvulsant medications should begin with the lowest possible dose followed by up titration. Level 1, Grade A.
		Hauser, W. et al. 2009 [17]	A meta-analysis of randomized controlled trials on treatment of fibromyalgia syndrome with gabapentin and PGB (6 RCT, 8733 participants) showed both drugs were associated wig small but significant pain reduction, improved sleep function but did not significantly affect the level of depression.	
		Moore, RA. et al. 2009 [16]	As Per EULAR	

**Table A3:** Cyclopnezaprine.

Guideline	No. of studies	Reviews	Comments	Recommendation
EULAR	1 review with 5 trials	Tofferi, J.K. et al. 2004 [40]	Systematic review including 5 placebo-control trials (*n* = 312). The odds ratio for global improvement was 3.0 (95% CI 1.6–5.6) with no improvement in fatigue or tender points in FM.	Weak for (75% agreement).
AWMF	List of studies included in the meta-analysis	Bennett, R.M. 1988 [75]	Double blind control study, comparison of cyclobenzaprine and placebo showed significant reduction in total tender points and fatigue.	Negative recommendation. strong consensus. Level of evidence 2a.
		Carette, S. et al. 1994 [55]	Randomised double blind trial to compare relative efficacy and safety of AMT and cyclobenzaprine. Confirm short-term efficacy and safety of both.	
		Hamaty, D. 1989 [76]	Double blind, cross-over study on plasma endorphin, prostaglandin and catecholamine profile of patients treated with cyclobenzaprine compared to placebo.	
		Reynolds, M. 1991 [77]	Double blind, placebo controlled cross-over design on the effects of cyclobenzaprine showed improvement in fatigue and total sleep with no benefit on pain.	

**Table A4:** SSRI.

Guideline	No. of Studies	Articles	Comment	Recommendation
EULAR	7 systematic reviews, 3 to 11 trials and up to 521 patients	Hauser, W. et al. 2012 [78]	Systematic review and meta-analysis including 18 RCTs on TCA, SSRI, SNRI and MAOI (*n* = 1427), concluded that antidepressant medications are associated with improvement in pain, depression, fatigue, sleep disturbance and health related QOL in FM.	Weak against (94%).
		Üçeyler, N. et al. 2008 [50]	Systematic review including 26 trials; AMT (13 RCTs), SSRIs (2 RCTs) and one on paroxetine. The SSRIs fluoxetine (20–40 mg/day), sertraline (50 mg/day), and paroxetine (20 mg/day) reduce pain and depressiveness and improve sleep and quality of life. Citalopram (20–40 mg/day) is not superior to placebo.	
		Häuser, W. et al. 2009 [65]	A meta-analysis on treatment of FMS with antidepressants, overall antidepressant are effective in FMS but the effect size of SSRIs was small.	
		Perrot, S. et al. 2014 [52]	Meta-analysis examining 6 core symptoms. Citalopram did not show any significant benefit on pain, fatigue or effective symptoms. Fluoxetine might be beneficial at high doses (80 mg).	
		Choy, E. et al. 2011 [66]	Systematic review and mixed treatment comparison including paroxetine and citalopram. Meta-analysis found no evidence of their superiority over placebo.	
		Jung, A.C. et al. 1997 [79]	Systematic review of RCTs on SSRIs (3 studies on FMS). SSRIs appear to be beneficial for mixed chronic pain, however it is unclear if SSRIs are beneficial for FMS.	
		Arnold, L.M. et al. 2000 [80]	Meta-analysis and review of antidepressants treatment of fibromyalgia including citalopram and fluoxetine did not show significant difference with placebo however only 2 trials were included for analysis.	
AWMF	Only articles used for met-analysis are included in this table	Arnold, L.M. et al 2002 [81]	Randomised, double blind placebo-control trial, on (*n* = 66), fluoxetine was found to be effective on most outcome measures and generally well tolerated in women with fibromyalgia	Serotonin reuptake inhibitors (SSRI; fluoxetine: 20–40 mg/day. paroxetine: 20–40 mg/day) can be considered for a limited time period in patients with co- morbid depressive disorder and anxiety disorder. EL 2a, open recommendation, consensus.
		Goldenberg, D. et al. 1996 [29]	Randomised double blind cross-over study, (*n* = 19) AMT 25 mg and Fluoxetine 20 mg. Both drugs are effective in FM and work better in combination.	
		Wolf, F. et al. 1994 [30]	Double blind placebo controlled trial, *n* = 42, fluoxetine 20 mg did not improve FM.	
		Nørregaard, J. et al. 1995 [82]	Double blind control trial, (*n* = 22)citalopram 20–40 mg. No benefit reported	
		Sencan, S. et al. 2004 [83]	Randomised control study, (*n* = 60) paroxetine 20 mg vs. aerobic exercise. Both modalities improve pain compared to placebo with no significant difference between the two interventions.	
		Anderberg, U.M. et al. 2000 [31]	Randomised, double blind (*n* = 40) placebo-control trial. Citalopram 20–40 mg. No significant change was found in the global judgment of improvement.	
		Patkar, A.A. et al. 2007 [84]	Randomised double blind placebo control trial (*n* = 116)on paroxetine (12.5–62.5 mg) showed that paroxetine improve overall symptomology in FMS.	
		Distler et al, 2010 [85]	Randomised double blind, placebo control parallel group to investigate the efficacy and safety of controlled release ropinirole (1–24 mg)	
Canadian	Reviews	Üceyler, N. et al. 2008 [50]	As per EULAR	All categories of antidepressants including SSRI may be used (level 1, grade A).
		Hauser, W. et al. 2009 [86]	Meta-analysis (as per EULAR)	

**Table A5:** Norepinephrine-Selective Reuptake Inhibitors (NSRI).

Guideline	No. of Studies	Articles	Comment	Recommendation
EULAR	DLX 8 systematic reviews including 2–6 trials, with 443 to 2249 participants Milnacipran 7 reviews, 1–5 trials, 125-4118 participants	Häuser, W. et al. 2011 [49]	Meta-analysis, ten AMT (*n* = 612), four DLX (*n* = 1411) and five MLN (*n* = 4129) studies, showed that 3 drugs were superior to placebo except DLX for fatigue, MLN for sleep and AMT for quality of life. AMT was superior to DLX and MLN for pain reduction, sleep disturbance and quality fif life improvement. DLX was superior to MLN in pain reduction, sleep disturbance and quality f life improvement. MLN was superior to DLX in reducing fatigue.	Milnacipran and Duloxetine weak for (100% agreement) Level of evidence 1A.
		Perrot, S. et al. 2014 [52]	Meta-analysis. DLX showed a statistically significant improvement in reducing pain, sleep disturbance, fatigue, affective symptoms, functional deficit and cognitive impairment. MLN improved pain but had no effect on sleep disturbance, fatigue, affective symptoms and functional deficit.	
		Häuser, W. et al. 2010 [21]	Review study on DLX, MLN and PGB (17 studies, *n* = 7739). The three drugs were superior to placebo for all outcomes. DLX and PGB were superior to MLN in reduction of pain and sleep disturbance, DLX was superior in reducing depressed mood, MLN and PGB were superior in reducing fatigue.	
		Choy, E. et al. 2011 [66]	A systematic review and mixed treatment comparison, confirmed the efficacy of PGB and SNRIs in treatments of FMS.	
		Häuser, W. et al. 2013 [27]	Cochrane review (*n* = 6038). 5 studied on Duloxetine, 5 studies on MLN. Both drugs provide a small benefit over placebo with no improvement in fatigue and quality of life (QOL).	
		Sultan, A. et al. 2008 [87]	Systematic review. DLX is equally effective for the treatment of peripheral diabetic neuropathy and FM. Doses higher than 60 mg do not provide additional pain relief, but cause slightly more withdrawal due adverse effects.	
		Lunn, M.P. et al. 2014 [22]	Cochrane review (*n* = 2249). Duloxetine in short-term (12 weeks) and long-term (28 weeks) is more effective than placebo at reducing pain (RR >30% pain, RR 1.38, 95% CI 1.22 to 1.56). There was no significant effect at 20–30 mg/day and no difference between 60 mg and 120 mg /day.	
		Ormseth, M.J. et al. 2010 [24]	Review article. MLN has a demonstrated efficacy in managing global FMS symptoms and pain at doses of 100 and 200 mg divided twice daily however it has numerous side effects.	
		Lunn, M.P. et al. 2014 [22]	Cochrane review on MLN for neuropathic pain in adults concluded that it was effective for a minority in the treatment of pain due to FM.	
		Derry, S. et al. 2012 [23]	Cochrane review on use of MLN for neuropathic pain (*n* = 4138) concluded that MLN is effective in a minority in treating pain in treating pain associated with FMS.	
AWMF	No systematic reviews. Duloxetine: 5 RCTs *n* = 1157 in the experimental group Milnacipran	Arnold, L.M. et al. 2004 [88]	Randomised double-blind, placebo controlled trial (*n* = 207). DLX (120 mg/day) is effective and safe in treating any symptoms of FM. 120 mg/day resulted in more than 50% pain reduction in 3 months.	Treatment with DLX (60 mg) can be considered for a limited time period in patients without comorbid depressive disorders or generalized anxiety disorders , if treatment with AMT is not possible. MLN should not be used. EL 1a, negative recommendation, consensus.
		Arnold, L.M. et al. 2005 [89]	Randomised double-blind placebo controlled trial. DLX 60 g, 90 mg and 120 mg were effective in treatment of fibromyalgia with or without depressive symptoms.	
		Arnold , L.M. et al. 2010a [90]	Randomised double blind placebo controlled trial, treatment with DLX 60, 90, 120 mg/day was associated with pain reduction, better sleep and quality of life.	
		Chappell, A.S. 2009 [91]	Randomised double blind placebo controlled trial. DLX 60/120 mg failed to develop significant improvement in the co-primary outcome measures (pain and patient global impression of improvement).	
		Russell, I.J. et al. 2008 [92]	Randomised double blind, placebo controlled trial (*n* = 1025) on MLN(100 mg/day vs. Placebo). MLN improved pain, global status, fatigue and physical and mental function.	
		Arnold, L.M. et al. (2010b) [93]	Randomised double blind placebo control trial. Milnacipran administered at a dosage of 100 mg/day improved pain, global status, fatigue, and physical and mental function in patients with fibromyalgia.	
		Branco, J.C. 2010 [94]	Randomised double blind, placebo controlled, multicentre clinical trial (*n* = 884). MLN 200 mg was associated with significant improvement.	
		Clauw, D.J. et al. 2008 [26]	A 15-week, multicentre, randomised, double-blind, placebo-controlled, multi-dose trial (*n* = 2270) of MLN 100 g, 200 mg and placebo showed significant improvement in both doses of MLN.	
		Mease, P.J. et al. 2009 [25]	Randomised double blind placebo controlled trial, on the efficacy and safety of MLN favored use of MLN in FM (*n* = 888).	
		Vitton, O. et al. 2004 [95]	Double blind placebo-controlled trial (*n* = 125). 75% of treated patients reported overall improvement compared to 38% in placebo group (*p* < 0.01). MLN may have the potential of relieving not only pain but several other symptoms in FM.	
Canadian		Üceyler, N. et al. 2008 [50]	Systematic review, 2 studies on DLX and 1 on Milnacipran. DLX 60–120 mg and MLN 25–500 mg/day reduce pain and depressive symptoms and improve sleep and quality if life.	Level 1 , Grade A All categories of antidepressants may be used for treatment of pain and other symptoms in fibromyalgia.
		Hauser, W. et al. 2009 [65]	Meta-analysis, 3 studies on DLX, 1 on MLN. Strong evidence for the efficacy of Duloxetine and MLN in reducing pain (smd, −0.36; 95% CI, −0.46 to −0.25; *p* < 0.001) and sleep disturbance. Strong evidence for DLX in improving depressed mood and quality of life.	

**Table A6:** Tramadol.

Guideline	No. of Studies	Studies	Comment	Recommendation
EULAR	2 reviews, 313–422 subjects	Roskell, N.S. et al. 2011 [70]	Meta-analysis. One study on Tramadol. Tramadol plus Paracetamol was more likely to achieve 30% improvement in pain (RR 1.77, 95% CI 1.26 to 2.48) (*n* = 313)	Weak for, 100% agreement.
		Choy, E. et al. 2011 [66]	Meta-analysis, only one study with tramadol (50–400 mg) was included which showed benefit compared to placebo	
AWMF		Bennett, R.M. et al. 2003 [35]	Double blind, randomised study with 313 participants. A tramadol/ acetaminophen was effective in treating FM.	Due to the limited data neither a positive nor a negative recommendation is possible for weak opioids. Strong consensus.
Canadian guidelines		Bennett, R.M. et al. 2003 [35]	As above	Trial of opioids (tramadol) for patients with moderate to severe pain , unresponsive to other treatment modalities level 2, grade D.
		Biasi, G. et al. 1998 [37]	Double blind cross-over experiment (*n* = 12) showed Tramadol to be beneficial in pain relief	

**Table A7:** Non Steroidal Anti-Inflammatory Drugs (NSAIDs).

Guideline	No. of Studies	Studies	Comment	Recommendation
EULAR	Systematic review of 2 clinical trials (*n* = 242)	Choy, E. et al. 2011 [66]	Systematic review, with no evidence of improved outcomes compared to placebo	Weak against (100% agreement).
AWMF	4 studies were identified however only 2 were included in meta-analysis	Russellm L.J. et al. 1991 [96]	Double blind placebo-controlled study with Ibuprofen and alprazolam. The two NSAIDs showed some benefit in FMS. (*n* = 78)	Nonsteroidal antirheumatics (NSAR) should not be used. EL 3a, negative recommendation, strong consensus.
		Quijada-carrera, J. et al. 1996 [97] (reported study outcome was only suitable for drop-out analysis)	Randomised double blind placebo controlled trial, (*n* = 164) showed that tenoxicam + bromazepan can be effective in some patients with FM however the improvement was no clinically nor statistically significant compared to placebo.	
		Yunus, MB, et al. 1989 [98]	Double blind placebo controlled trial on ibuprofen(*n* = 46) did not show any benefit compared to placebo.	
Canadian guideline 2012		Rao, SG. et al. 2004 [99]	As fibromyalgia is a central pain syndrome, classes of drugs such as NSAIDs and opioids which act peripherally are ineffective.	In the event that NSAIDs are prescribed, particularly with associated conditions, they should be used for the shortest period of time and lowest possible dose. (Level 5 grade D).
		Tannenbaum, H. et al. 2006 [100]	An evidence-based approach to prescribing NSAIDs drugs.	
